# Searching for carbonylome biomarkers of aging – development and validation of the proteomic method for quantification of carbonylated protein in human plasma

**DOI:** 10.3325/cmj.2020.61.119

**Published:** 2020-04

**Authors:** Sanja Radman, Sanda Raić, Ivona Bućan, Ajka Pribisalić, Josipa Dunatov, Ivana Mudnić, Mladen Boban, Francois Xavier Pellay, Ivana Kolčić, Ozren Polašek

**Affiliations:** 1 Mediterranean Institute for Life Science, Split, Croatia; 2 University of Split School of Medicine, Split, Croatia; The first three authors contributed equally.

## Abstract

**Aim:**

To develop a method for measuring protein carbonylation in human plasma and serum samples, which was previously implied in numerous age-related phenotypes.

**Methods:**

Protein expression and carbonylation were analyzed in plasma samples obtained from 12 healthy human individuals by using a novel method that combines affinity-based albumin and immunoglobulin G removal, and aminooxy dyeing in one- or two-dimensional gels. In addition, carbonylome profile of plasma and serum was compared. Coefficients of variation and intra-class correlation coefficients were used in statistical analysis.

**Results:**

Following a step-wise laboratory development and optimization process, we measured the protein expression and carbonylation for 813 proteins from the plasma. The analysis of repeated measurements suggested excellent coefficients of variation, which rarely exceeded 10%. The average value of intra-class correlation based on absolute agreement (ICC) for protein expression was 0.97 ± 0.02, while for carbonylation it was 0.73 ± 0.24. The removal of the most extreme protein outlier in carbonylation assessment increased the average ICC to 0.87 ± 0.04. Low protein spot volume substantially reduced repeatability. Serum carbonylation estimates were similar to those from plasma, with the ICC in the range of 0.86-0.89.

**Conclusion:**

We developed a reliable method for the measurement of human plasma protein carbonylation, which can be used for the assessment of carbonylome biomarkers of aging.

Protein oxidation was systematically shown to change across age and age-related diseases, including Alzheimer’s disease, Huntington’s disease, amyotrophic lateral sclerosis, and various cancers (1,2). As organism ages, its molecules accumulate oxidative damage from reactive co-products of respiration and other metabolic processes. Although oxidation occurs in all biomolecules, proteins seem to be the most attractive research target due to their key role in biological processes. A very interesting form of oxidation is carbonylation (3), in which side chains of lysine, arginine, proline, and threonine are oxidized (4). It is an irreversible oxidative damage, which often leads to a loss of protein function and is supposed to be prevalent in severe oxidative damage and disease-related protein dysfunction (5). Despite rather high interest and favorable theoretical background, the measurement of carbonylation is plagued by numerous methodological problems, including the selection of labeling agents, samples, protocol, and low repeatability (6,7).

Two important methods substantially improved this field of research. The first is the use of fluorescence component (8-11), including CyDye fluor dyes (Cy2, Cy3, and Cy5). Besides having the same size and charge, they are pH insensitive, spectrally resolvable, highly sensitive, and bright and rather photo-stable, and most importantly, they do not affect the mass spectrometry results (12). The second important method was the development of difference gel electrophoresis (DIGE), which quantifies each protein as a single spot, reduces gel-gel variability via concomitant analysis of multiple samples (13), and yields much better repeatability (14,15).

Blood plasma, due to its accessibility, is one of the most common sources of information in clinical medicine. It is also one of the most diverse sources of protein, which contains blood proteins, as well as proteins secreted from other tissues (16). At the same time, it is a very complex mixture, and diversity and possibility for protein aggregation may present methodological problems in quantification (17,18). Nevertheless, the search for previously published articles did not suggest the existing method of measuring carbonylation from plasma and serum samples using DIGE.

The aim of this study was to develop a reliable method for measuring carbonylation from human plasma and serum samples, with a focus on the development of a robust biomarker for aging, which can be used in large-scale biobanking.

## Materials and methods

### Participant selection

The participants were selected from the project 10 001 Dalmatians (19-22) or the research program BioWine (23). In order to be considered for enrollment, the participants had to be healthy (no history of chronic disease or medication). Additionally they underwent a series of clinically relevant measurements to confirm their health status. This included a complete blood count and basic set of biochemical measurements, ECG, spirometry, blood pressure measurement, blood vessel Doppler analysis (ankle-brachial pressure index), and central arterial pressure and augmentation index measurement by Sphygmocor (AtCor Medical, West Ryde, Australia). All participants signed the informed consent before they entered the study, and the study was approved by the Ethics Committee of the Medical School, University of Split (003-08/13-03/0003 and 003-08/11-03/0005).

### Plasma and serum specimens

The study focused on plasma samples, which were obtained from peripheral venous blood samples collected in fasting conditions with the Sarstedt EDTA tube (10 mL Monovette, Sarstedt, Newton, NC, USA). The same provider was also used in the case of the serum samples (10 mL Monovette). The samples were centrifuged at 4°C for 10 min at 2000 g. The supernatants were centrifuged for the second time, and the isolated plasma was immediately transferred into a clean tube in aliquots (200-500 μL). Immediately upon collection and separation, all serum and plasma aliquots were frozen at -80°C to allow for the single-batch analysis of the entire set.

### Excessive protein removal

In order to deplete highly abundant proteins, mostly albumin and immunoglobulin G (IgG), we used a multiple affinity removal column HSA/IgG, 4.6 × 50 mm (Agilent Technologies, Santa Clara, CA, USA). An aliquot of 100 μL of plasma was diluted with 300 μL of manufacturer’s buffer A (Agilent Technologies), containing protease inhibitor mix to a final volume of 400 μL and filtered through 0.22 μm spin-X centrifuge tube filter (Costar, Washington, DC, USA) for 1 min at 16 000 g. The manufacturer’s instructions were followed for system and column conditioning, and 100 μL of sample was injected at a flow rate of 0.25 mL/min of buffer A. Flow-through fraction from 1.25 to 9 min (λ = 280 nm), containing low abundant proteins, was collected and buffer exchange was performed using Amicon Ultra centrifugal filter devices with 3 kDa cut-off (Merck Millipore, Burlington, MS, USA).

The first step of buffer exchange was performed in 4 mL Amicon Ultra centrifugal filter devices, where approximately 2 mL of collected flow-through fraction PBS buffer (1X PBS + PI) were added to a final volume of 4 mL. Centrifugation was performed on 4°C for 40 min at 4000 g, after which a sample was collected and placed to a 0.5 mL Amicon Ultra centrifugal devices with 3 kDa cut-off. PBS buffer was added to a total volume of 500 μL and centrifuged at 4°C for 30 min at 14 000 g. This step was repeated 3 times, after which a concentrated sample was collected and stored at -80°C until further analysis.

### Carbonyls labeling

Stock solution of 5 mM CF®647DI-aminooxy dye (CF, Biotium, Fremont, CA, USA) was prepared by adding 322.2 μL of DMSO to 1 mg of dye (Sigma, St. Louis, MO, USA). Sample concentration was measured by using Bradford reagent (Sigma), and the appropriate amount of sample was diluted with 1X PBS buffer. The first optimization step was to adjust the necessary amount of dye for carbonyl labeling. A 50-μmolar equivalent of CF aminooxy dye was added to the prepared sample as a control sample for labeling amount adjustment. To other samples we added 95%, 80%, 50%, 20%, 10%, 5%, and 1% of CF aminooxy dye volume added to control sample. The ligation was initiated by adding 1/10 volume of aniline acetate catalyst. The samples were stained overnight at 4°C and 600 rpm to ensure the labeling of all aldehydes and ketones. The samples were applied on the SDS-PAGE, which consisted of 12.5% resolving and 5% stacking gel. Electrophoresis was carried out at 75 V for 30 min, after which the current was increased to 150 V. The gels were scanned with a Typhoon Scanner (GE Healthcare, Chicago, IL, USA), and images were processed with TotalLab Quant (version 13.2, TotalLab, Saint Helens, UK).

### Selection of the SDS-PAGE percentage

To optimize the percentage of SDS-PAGE, 10%, 12.5%, and 15% resolving gel was prepared with 5% stacking gel. Before loading plasma biological replicates on the gels, 4x Laemmli SDS electrophoresis buffer (8% SDS, 4% 2-mercaptoethanol, 35% glycerol, 80% BPB, 150 mM Tris HCl pH 6.8) was added to the samples. Electrophoresis was carried out at 75 V for 30 min, after which the current was increased to 150 V. To determine total protein expression, gels were stained with Serva purple dye. The first step was to fix the gels in 25-50 mL of fixation solution (1% citric acid, 15% ethanol) for 15 min with gentle rocking. After this, the gels were placed into the staining solution with Serva purple, which was prepared fresh (1 V of Serva purple in 250 V of staining solution: 0.1 M boric acid, 0.1 M NaOH; Serva, Heidelberg, Germany) with gentle rocking for 10 min. The gels were then washed in the washing solution (15% ethanol) with gentle rocking for 10 min and finally acidified with gentle rocking for 10 min in fixation solution. The gels were scanned with a Typhoon Scanner, and images were processed with TotalLab Quant (version 13.2, TotalLab).

### 2D electrophoresis

We used labeling CF aminooxy and Cy3 dyes (Lumiprobe, Hannover, Germany), once containing 1X PBS buffer and once containing UTC buffer (7 M urea, 2 M thiourea, 2% CHAPS, 40 mM DTT, 0.5% IPG buffer). After labeling the carbonyls and before the labeling with Cy3, the pH of the samples was adjusted to 8.5. Internal standard labeled with Cy2 (in 1X PBS or UTC) was added to the sample labeled with CF aminooxy dye and Cy3. Rehydration buffer A (7M urea, 2M thiourea, 2% CHAPS, 60mM DTT, 0.5% IPG buffer, 0.002% BPB) was added to the pooled sample to a total volume per strip of 450 μL and placed into an IPGbox (GE health care). IPG strips pH 4-7 length 24 cm (GE Healthcare) were carefully placed with gel facing down, and the liquid was distributed evenly under the strip. The IPGbox lid was closed, and IPG strips were left to rehydrate at room temperature overnight.

The first dimension, isoelectric focusing, was carried out in Ettan IPGphor 3 isoelectric focusing system (GE Healthcare). The second dimension was carried out in the Ettan DALTsix electrophoresis system (GE Healthcare). IPG strips were placed at the top of a 10% SDS-PAGE gel and overlaid with agarose sealing solution (25 mM Tris base, 192 mM glycine, 0.1% SDS, 0.5% agarose, 0.002% BPB). The electrophoresis was performed at 25°C in two steps: at 10 mA/gel for 1 h and at 12mA/gel until the moment the tracking dye leaked out from the gels. Gels were scanned with a Typhoon Scanner, and the images were analyzed with SameSpots software (TotalLab). Finally, rehydration was achieved by the rehydration buffer B (7M Urea, 2M Thiourea, 4% CHAPS, 40mM DTT, 1% IPG buffer, 0.002% BPB; Agilent Technologies). The summarized scheme of the original and modified protocol is provided in the Supplementary Table 1.(Supplementary Table 1)

### Statistical analysis

The analytic part of this study was based on the repeatability analysis, which was performed with three methods. First, we calculated the coefficients of variation (CoV) or relative standard deviation, defined as the ratio of standard deviation and mean for any set of repeated measurements. After CoV calculation for every protein spot, we averaged all values to get an average individual CoV. Second, we calculated intra-class correlation coefficients (ICC), based on the two-way random, means-focused, absolute agreement approach, which focuses on reliability analysis (24). In order for our results to be comparable with similar studies, we also provided Cronbach’s α estimates. Plasma and serum samples were compared by using Bland-Altman analysis, as they are two different biological samples from the same participant. The coefficients of variability and protein spot volume were correlated by using Spearman’s rank correlation test. The protein spots that were in disagreement in plasma and serum were analyzed by using *t* test. The data were analyzed in SPSS, version 21 (IBM, Armonk, NY, USA), with the level of significance set at *P* < 0.05. The entire raw data set is provided in the Supplementary Material.

## Results

### Measurement method optimization

During the protocol development, we examined how changes in critical steps affected the results. First, we defined the minimum amount of aminooxy in order to achieve the least cost and least possible processing contamination. The use of only 20% of the amount suggested by the manufacturer resulted in a successful labeling of 98% of all carbonyls, and the use of 10% resulted in 87% of labeled carbonyls. For one-dimensional gels we used 0.2 μL of CF®647DI aminooxy to 15 μg of proteins (corresponding to 13% of the suggested amount) and for two-dimensional gels we used 70 μg of proteins (corresponding to 17% of the suggested amount), suggesting a possible substantial reduction in comparison with the original protocol (Supplementary Figure 1(Supplementary Figure 1)).

We also aimed to examine how resolving gel on SDS-PAGE affected the results. We used 10%, 12.5%, and 15% of resolving gel, with the best results obtained in 12.5% resolving gel with 5% stacking gel (Supplementary Figure 2(Supplementary Figure 2)). Another step included the use of the optimal buffer with aminooxy dye. The PBS buffer was better than UTC for Cy3 dye (Supplementary Figure 3(Supplementary Figure 3)). PBS buffer outperformed UTC even in the case of aminooxy; 868 distinct protein spots were observed in PBS buffer, compared with 779 spots in UTC buffer (Supplementary Figure 4(Supplementary Figure 4)).

The adherence to the suggested IEF protocol did not yield any usable results (possibly due to the existence of naturally occurring NaCl in plasma). Therefore, we performed numerous mini-modifications in order to get a meaningful output. This resulted in the use of modified rehydration buffer and some deviations from the IEF protocol, mainly in electrophoresis duration, voltage, and type (Supplementary Table 1(Supplementary Table 1)).

### Method repeatability analysis

The measurements of plasma samples of 12 healthy participants yielded 813 detectable protein expression and carbonylation spots. The repeatability of protein expression data was excellent, with CoV that usually did not exceed 10% (Table 1). The CoV for carbonylation were slightly higher (Table 1), which can be explained by the dependency of protein carbonylation to protein amount and subsequent additivity of variability. After the analysis of the least repeatable protein spots, we observed a substantial outlier. When we removed this spot (protein spot 355), the ICC increased (Table 1).

**Table 1:** Coefficients of variation (CoV) and intra-class correlation coefficients (ICC; two-way mixed, consistency measure) and Cronbach’s alpha for protein expression and carbonylation in 12 analyzed participants

Individual (age, sex)	Protein expression			Protein carbonylation (with protein spot 355 removed)		
	coefficients of variation; mean ± standard deviation (min-max)	ICC (95% confidence interval)	α*	coefficients of variation; mean ± standard deviation (min-max)	ICC (95% confidence interval)	α*
CNXHS35 (45 years, female)	11.0 ± 7.9 (0.3-50.0)	0.938 (0.931-0.945)	0.938	11.1 ± 7.5 (0.5-73.0)	0.760 (0.621-0.836)	0.812
CNXHS46 (64, female)	8.2 ± 6.5 (0.1-109.6)	0.972 (0.968-0.975)	0.972	8.2 ± 7.6 (0.2-164.9)	0.862 (0.843-0.879)	0.866
CNXHS54 (32, male)	6.8 ± 4.8 (0.1-39.7)	0.982 (0.980-0.984)	0.982	9.2 ± 5.8 (0.1-47.0)	0.819 (0.684-0.884)	0.866
CNX1519 (65, female)	8.4 ± 5.0 (0.3-47.1)	0.979 (0.977-0.982)	0.979	8.1 ± 4.9 (0.7-50.4)	0.880 (0.863-0.895)	0.886
CNX0941 (45, female)	8.6 ± 5.8 (0.7-62.8)	0.976 (0.974-0.979)	0.976	9.2 ± 5.4 (0.5-81.4)	0.806 (0.773-0.834)	0.820
CNX1012 (58, female)	9.1 ± 5.7 (0.6-84.4)	0.977 (0.975-0.980)	0.977	9.3 ± 5.7 (0.7-93.0)	0.760 (0.732-0.786)	0.764
CNXWS37 (26, male)	7.8 ± 4.6 (0.3-29.4)	0.983 (0.980-0.984)	0.983	7.2 ± 4.8 (0.7-43.9)	0.906 (0.895-0.916)	0.907
CNXWS41 (24, male)	9.2 ± 6.5 (0.7-101.8)	0.975 (0.971-0.978)	0.976	8.1 ± 6.2 (0.8-114.4)	0.893 (0.880-0.904)	0.894
CNXWS45 (30, male)	8.4 ± 5.6 (0.7-49.7)	0.979 (0.975-0.983)	0.981	8.0 ± 5.4 (0.3-66.0)	0.893 (0.879-0.906)	0.897
CNXWS49 (28, male)	8.4 ± 5.7 (0.9-42.0)	0.980 (0.977-0.982)	0.981	7.4 ± 5.1 (0.6-68.3)	0.897 (0.885-0.908)	0.898
CNXWS53 (26, male)	9.4 ± 6.3 (0.8-95.1)	0.977 (0.966-0.983)	0.981	7.9 ± 5.4 (0.3-78.7)	0.888 (0.867-0.905)	0.897
CNXWS57 (26, male)	7.3 ± 6.5 (0.5-141.7)	0.986 (0.985-0.988)	0.986	7.0 ± 11.8 (0.4-322.8)	0.914 (0.904-0.924)	0.916

*Cronbach’s alpha.

The most obvious cause for lower repeatability of some protein spots was the spot volume, which reflects the total amount of protein in each spot (r = -0.846; *P* < 0.001). The average value of CoV steadily decreased with the increase in spot volume (Supplementary Table 2(Supplementary Table 2)).

The comparison of plasma and serum results for three participants provided reasonably reliable estimates, with CoV that varied from 5.05 to 5.82 and ICC that varied from 0.863 to 0.893. The Bland-Altman analysis suggested 22 outliers that were out of limits of agreement (Supplementary Figures 5-7(Supplementary Figures 5-7)). The disagreement between plasma and serum was not associated with protein spot volume (*P* = 0.376).

## Discussion

This study describes a protocol for measurement of plasma protein carbonylation, which was shown to have very good repeatability, slightly lower than protein expression. Good repeatability is not novel for DIGE (14), but we did not find a previous study that investigated the repeatability of carbonylation measurement. As expected, variation was higher when the abundance of protein was very low, ie, too close to the detection limit, affecting protein carbonylation even more due to its dependency on expression. Although this is not the first attempt to measure carbonylated protein from human plasma (25,26), our method provides a wider protein coverage and more repeatable results.

The protein spot volume was the main factor that defined the repeatability of carbonylation estimates. While this finding is intuitive, the disagreement in plasma vs serum was seemingly different, and did not depend on this. This indicates that the disagreement is more likely due to different protein composition between plasma and serum. A definitive answer can be provided by the identification of the proteins. Nevertheless, this study shows that plasma and serum can be used more or less interchangeably in carbonylation assessment.

One of the main limitations of the proposed method is that it is only able to quantify the carbonylation signal, without having the ability to identify the protein in the spot. This step is usually performed in the mass spectrometry or a similar method for identification. This is the main reason why we referred to the results as spots, since we were not able to identify these spots. Therefore, the next stage of the method development is to integrate it with one of such methods, in order to facilitate its biological validation and further progression toward a possible biomarker discovery.

A growing number of methods and approaches that assess biomarkers of aging are being tested. The multidimensionality of aging and its nonlinear nature (27) will surely require the refinement of multi-molecular biomarkers, which will reflect different (patho-)biological processes across different stages of life. In addition, the use of multiple biomarker types will enable the analysis of their possible synergy or understanding of shared variance, which can further contribute to our understanding of aging but also improve the predictive value of biomarkers. This is where the existing long-term cohort biobanks are coming into the focus of science of aging (28), since they offer a quick and easily accessible source of life-long data.
