# Supplementary material for: Searching for carbonylome biomarkers of aging – development and validation of the proteomic method for quantification of carbonylated protein in human plasma
Source: Croat Med J. 2020 Apr;61(2):119–25. doi: 10.3325/cmj.2020.61.119 (PMC7230409; doi:10.3325/cmj.2020.61.119)
Supplement: Supplementary Table 1 [file CroatMedJ_61_s001.pdf]

Supplementary Table 1. Iso-electric focusing protocol (original) and modified

**Parameter**	**Original protocol**	**Modified protocol**
Temperature (°C)	20	20
Current (µA)	50	50
Step 1 (step & hold)	500 V, 7 h, 3.5 kVh	30 V, 12 h, 0.4 kVh
Step 2 (gradient)	1000 V, 1h, 0.8 kVh	500 V, 1h, 0.3 kVh
Step 3 (gradient)	8000 V, 3h, 13.5 kVh	1000 V, 1h, 0.8 kVh
Step 4	8000 V, 5:16 h, 42.4 kVh (step & hold)	8000 V, 1h, 4.5 kVh (gradient)
Step 5	-	8000 V, 7:30h, 60.0 kVh (step & hold)
Total time (h)	18	22:30
Energy (kVh)	60	65.9
