# Supplementary material for: Searching for carbonylome biomarkers of aging – development and validation of the proteomic method for quantification of carbonylated protein in human plasma
Source: Croat Med J. 2020 Apr;61(2):119–25. doi: 10.3325/cmj.2020.61.119 (PMC7230409; doi:10.3325/cmj.2020.61.119)

Supplementary Figure 1. One-dimensional gel showing a comparison of aminooxy dye amount for total protein carbonylation. Control sample (100%) and 95%, 80%, 50%, 20%, 10%, 5% and 1% of CF aminooxy dye volume added to control sample.

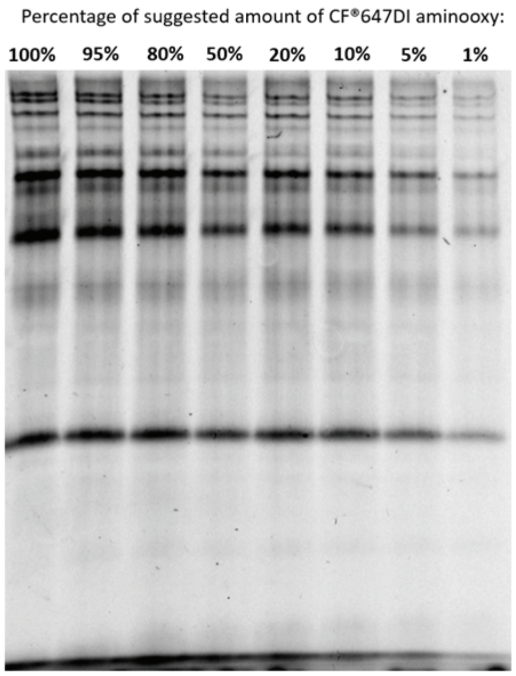

Supplement: Supplementary Figure 1 [file CroatMedJ_61_s002.pdf]
