# Supplementary figures and images for: Searching for carbonylome biomarkers of aging – development and validation of the proteomic method for quantification of carbonylated protein in human plasma
Source: Croat Med J. 2020 Apr;61(2):119–25. doi: 10.3325/cmj.2020.61.119 (PMC7230409; doi:10.3325/cmj.2020.61.119)

Supplementary Figure 2. One-dimensional gel showing a scan of carbonyls on a) 10%, b) 12.5 % and c) 15% resolving gel

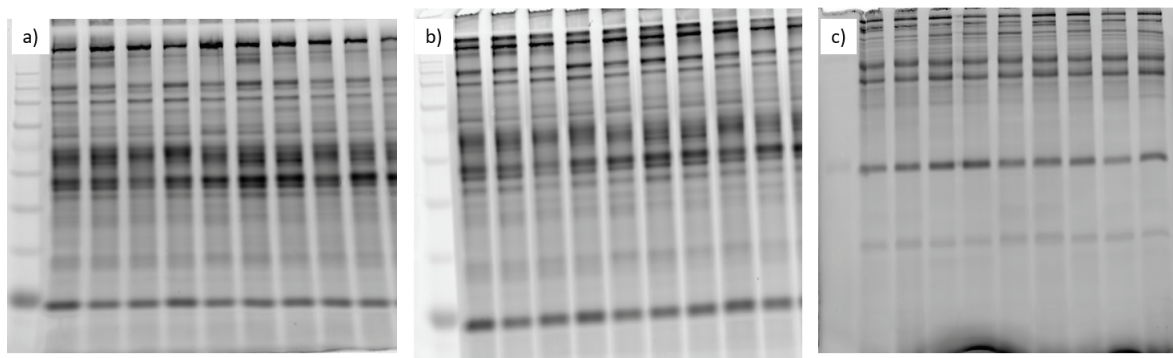

Supplement: Supplementary Figure 2 [file CroatMedJ_61_s003.pdf]

Supplementary Figure 3. Two-dimensional gel labelled with Cy3, a) PBS buffer, b) UTC buffer

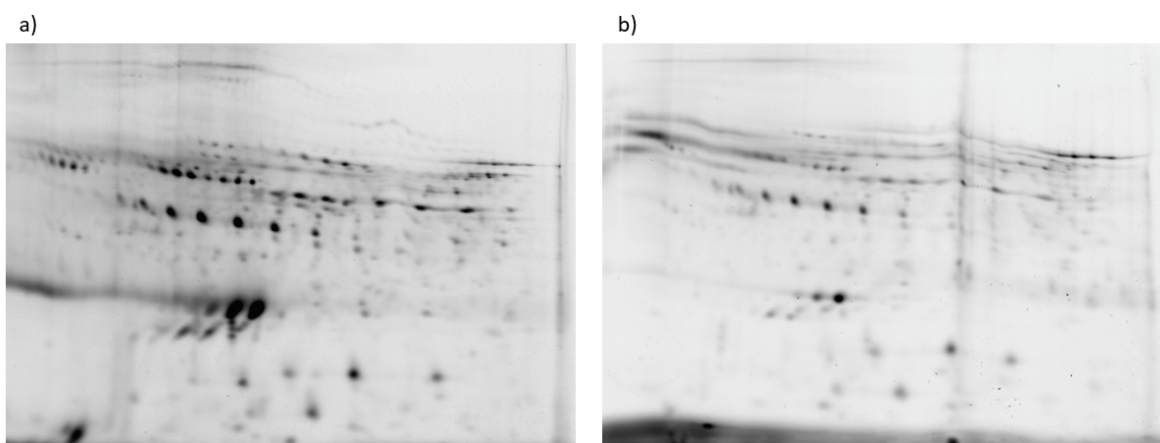

Supplement: Supplementary Figure 3 [file CroatMedJ_61_s004.pdf]

Supplementary Figure 4. Two-dimensional gel labelled with aminooxy, a) PBS buffer, b) UTC buffer

a)

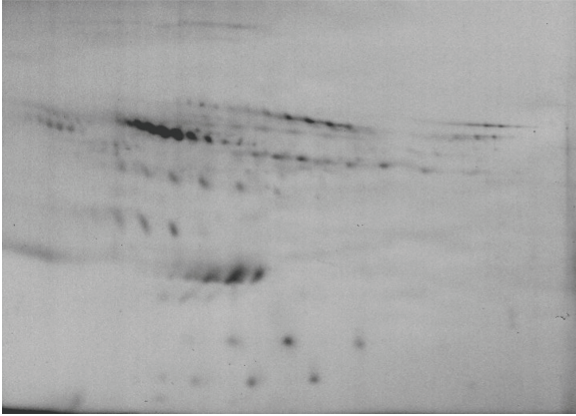

b)

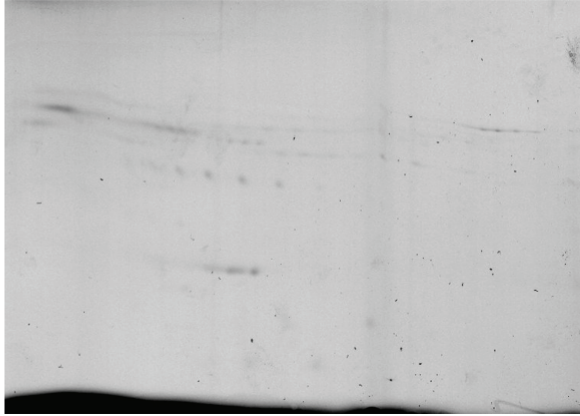

Supplement: Supplementary Figure 4 [file CroatMedJ_61_s005.pdf]
