# Supplementary material for: Searching for carbonylome biomarkers of aging – development and validation of the proteomic method for quantification of carbonylated protein in human plasma
Source: Croat Med J. 2020 Apr;61(2):119–25. doi: 10.3325/cmj.2020.61.119 (PMC7230409; doi:10.3325/cmj.2020.61.119)
Supplement: Supplementary Table 2 [file CroatMedJ_61_s006.pdf]

Supplementary Table 2. Comparison of the cut-off value of protein volume and number of retained proteins and their coefficients of variation

Spot volume cut-off	Number of retained protein spots	Average individual CoV±SD
All proteins	813	8.39±4.42
50	799	8.12±2.75
100	732	7.66±1.11
150	667	7.32±2.04
200	606	7.05±1.87
250	562	6.89±1.79
500	420	6.35±1.57
1000	220	5.66±1.37
2000	86	5.07±1.37
