# Supplementary material for: Searching for carbonylome biomarkers of aging – development and validation of the proteomic method for quantification of carbonylated protein in human plasma
Source: Croat Med J. 2020 Apr;61(2):119–25. doi: 10.3325/cmj.2020.61.119 (PMC7230409; doi:10.3325/cmj.2020.61.119)

Supplementary Figure 5. Bland-Altman graph of relationship between plasma and serum carbonylation, subject CNX1519

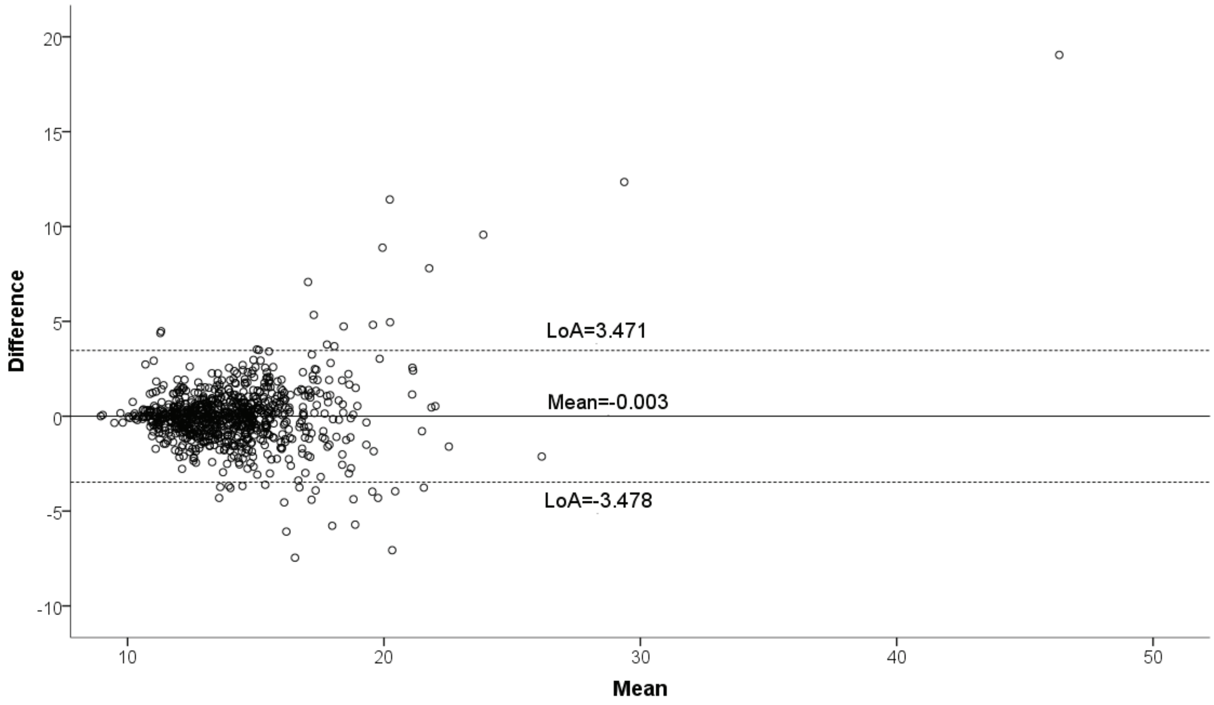

Supplementary Figure 6. Bland-Altman graph of relationship between plasma and serum carbonylation, subject CNX1012

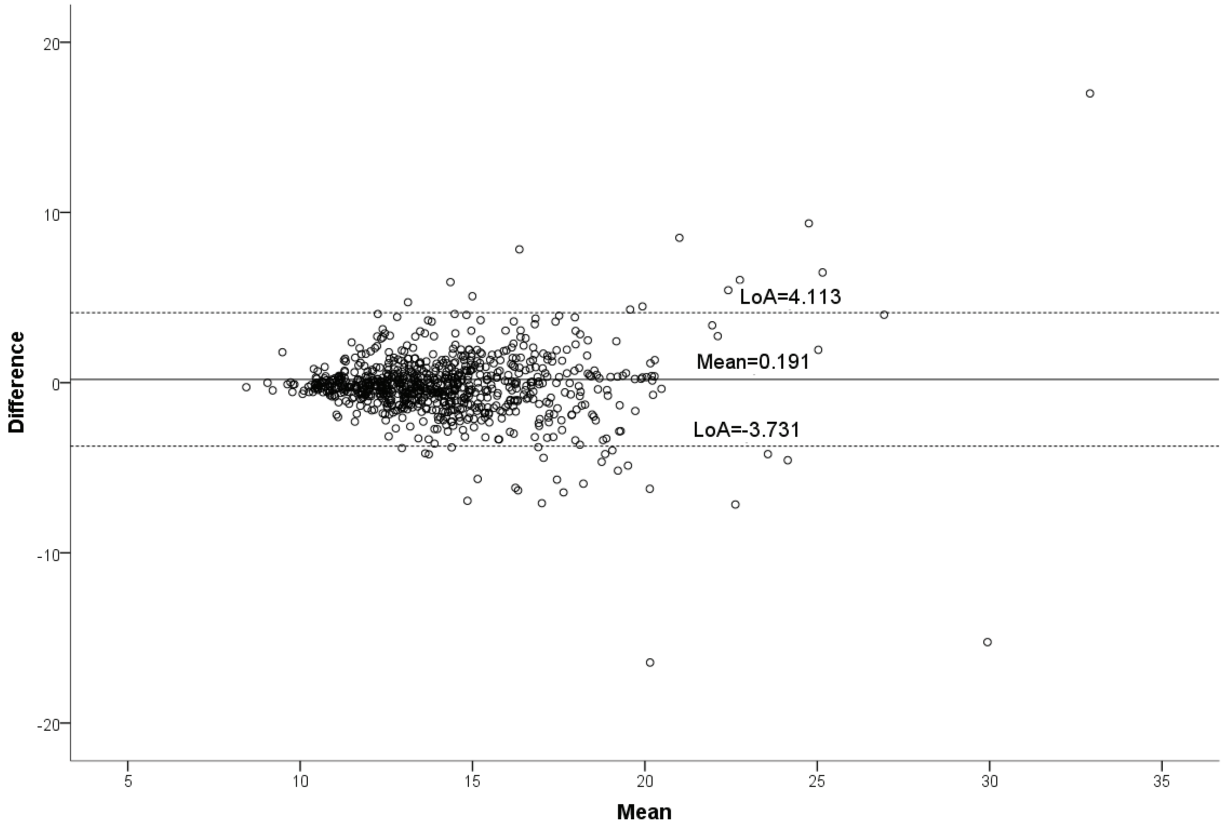

Supplementary Figure 7. Bland-Altman graph of relationship between plasma and serum carbonylation, subject CNX0941

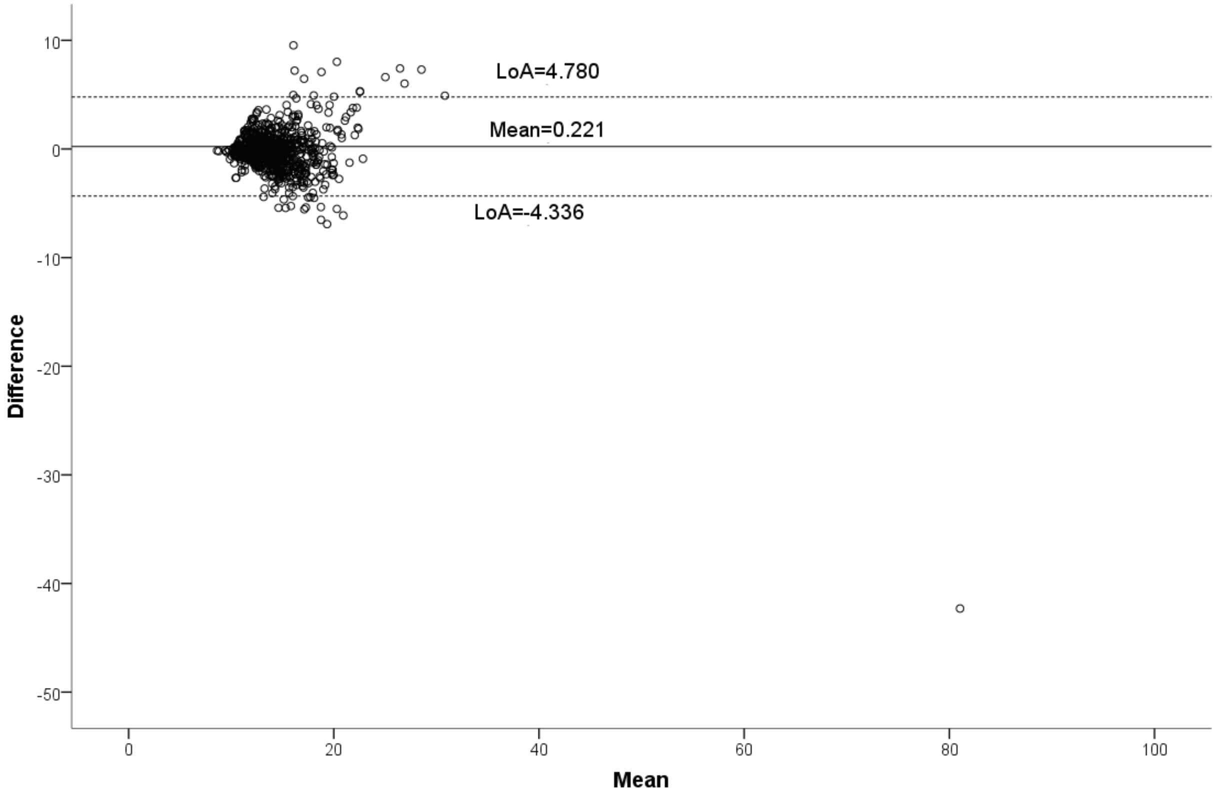

Supplement: Supplementary Figures 5-7 [file CroatMedJ_61_s007.pdf]
